# Association between digestive symptoms and sleep disturbance: a cross-sectional community-based study

**DOI:** 10.1186/s12876-019-0945-9

**Published:** 2019-02-19

**Authors:** Min Kyung Hyun, Younghwa Baek, Siwoo Lee

**Affiliations:** 1 Department of Preventive Medicine, College of Korean Medicine, Dongguk University, Gyeongju, 38066 Republic of Korea; 2 Future Medicine Division, Korea Institute of Oriental Medicine, Daejeon, 1672 Republic of Korea

**Keywords:** Digestive symptoms, Sleep disturbances, The Korean Genome and Epidemiology Study_Ansan and Ansung cohort, The Korean Medicine Data Center

## Abstract

**Background:**

This study was conducted to analyze the association between digestive symptoms and sleep disturbance, and to determine if any digestive symptoms are related to sleep disturbance.

**Methods:**

This was a cross-sectional study of 5792 subjects surveyed in a community-based cohort. Subjects provided information regarding the quality of sleep as assessed by the Pittsburgh Sleep Quality Index (PSQI), as well as digestive symptoms as assessed by the Gastrointestinal Symptom Rating Scale (GSRS). Logistic regressions were used to examine factors associated with sleep quality.

**Results:**

The mean PSQI global score of the no sleep disturbances group (*n* = 4948) was 3.92 (SD = 2.14), while that of the sleep disturbance group (*n* = 844) was 11.18 (SD = 2.17). The association between digestive symptoms and sleep disturbance was evaluated by logistic regression after adjusting for cofounding factors. The results revealed that sleep disturbances were associated with digestive symptoms (aOR = 1.29, 95% CI = 1.22–1.36), especially abdominal pains (aOR = 1.63, 95% CI = 1.19–2.25), acid regurgitation (aOR = 1.48, 95% CI = 1.17–1.86), abdominal distension (aOR = 1.80, 95% CI = 1.42–2.28), and eructation (aOR = 1.59, 95% CI = 1.24–2.03).

**Conclusions:**

Digestive symptoms and sleep disturbances seem to be associated. These results will help medical professionals to effectively diagnose and manage patients with sleep disturbance. Furthermore, subsequent studies using comprehensive longitudinal data should be conducted to confirm the results of the present study.

## Background

Sleep disturbance encompasses various sleep disorders including insomnia, hypersomnia, circadian rhythm sleep-wake disorders, sleep apnea, narcolepsy and cataplexy, parasomnia and sleep related movement disorders [1, 2].

Many people experience sleep disturbances worldwide, the most common of which is insomnia [3]. According to the American Psychiatric Association, 10–20% of people complain of significant sleep problems to their primary care physician [4]. Moreover, 16.6% of adults aged 50 years or older reported having nocturnal sleep problems in a prior study [5]. The finding that many older adults are commonly dissatisfied with sleep quality and report sleep-related diseases indicates that the prevalence of sleep disturbances increases as the population ages [6].

These sleep disturbances are associated with the occurrence of various diseases. Specifically, it has been reported that sleep disturbances may be associated with psoriasis, suicidal ideation, alcohol use disorders, mental health difficulties, and nocturnal acid reflux [7–10]. There have also been reports of sleep disturbances being associated with functional gastrointestinal disorders (FGID). In a systematic review of patients with irritable bowel syndrome (IBS), there was evidence of a positive correlation between subjectively, but not objectively, reported sleep disturbances and gastrointestinal symptom exacerbation among people with IBS [11]. In a study of 3600 Chinese patients, FGID were significantly associated with excessive daytime sleepiness [12]. Moreover, a 2002 study of Japanese people who underwent annual health checkups at a hospital revealed positive associations between gastroesophageal reflux disease and sleep disturbances [13]. Furthermore, a population-based study of 25,844 Norwegians revealed that sleep disturbances and gastroesophageal reflux symptoms (GERS) were bidirectionally associated, suggesting that sleep disturbances may lead to GERS and vice versa [14].

In the Neijing, which is an ancient oriental medical text, stomach disharmony is the major cause of the sleep disturbance [15]. Although stomach disharmony is a general term referring to various functional disorders of the stomach [16], it is uncertain if specific digestive symptoms are related to stomach disharmony.

Despite studies conducted to date, there is a lack of research regarding the relationship between sleep disturbances and digestive symptoms using large population datasets. Furthermore, we are aware of no studies that have examined the interaction between sleep disturbances and specific digestive symptoms. Therefore, this community-based study was conducted to: (a) investigate the association between digestive symptoms and sleep disturbance, and (b) analyze whether any digestive symptoms are related to sleep disturbance.

## Methods

### Study design and data source

We conducted a cross-sectional analysis of the community-based cohort survey of the Korean Medicine Data Center (KDC) that was conducted from June 2012 to December 2014. The KDC is a consortium project consisting of hospital, clinic, and community-based cohort data collected in the Republic of Korea and hospital data collected in Japan, China, Vietnam and the United States of America [17–19]. The KDC community-based cohort data was collected as part of a follow-up survey of the “The Korean Genome and Epidemiology Study (KoGES)_Ansan and Ansung cohort,” which is an ongoing, community-based cohort study that started in 2001 [17, 20]. The participants in the KoGES_Ansan and Ansung cohort were residents aged 40 to 69 who were extracted by two-stage cluster sampling based on gender, age distribution, and information regarding the governing district obtained in the 2000 census [21].

These survey data were collected through face-to-face interviews with participants using structured questionnaires after the interviewers were trained according to a pre-established protocol. During interviews, information was obtained regarding the participants’ characteristics, results of physical examinations and laboratory analyses, symptoms, etc.

### Variables definition

#### Sleep disturbances

The quality and disturbance of sleep was evaluated by the PSQI, a self-administered questionnaire that assesses usual sleep habits during the previous month [22, 23]. The individual items generate seven component scores pertaining to subjective sleep quality, sleep latency, sleep duration, habitual sleep efficiency, sleep disturbances, use of sleeping medicine, and daytime dysfunction [22]. The sum of these seven components yields a global PSQI score that ranges from 0 to 21 [22]. The PSQI is useful for distinguishing between sleep disorders and good sleepers. The cutoff score of the original English version of the PSQI is 5 points and the diagnostic sensitivity and specificity of good or poor sleepers are 89.6 and 86.5%, respectively [22]. However, the cutoff score of the Korean version of the PSQI is 8.5, and the diagnostic sensitivity and specificity are 94.3 and 84.4%, respectively [23]. Therefore, we defined a PSQI of 8.5 or more as having sleep disturbance.

#### Digestive symptoms

The digestive conditions of each subject were assessed by the digestion component portion of the Gastrointestinal Symptom Rating Scale (GSRS) [24, 25]. The GSRS is a disease specific instrument consisting of 15 items combined into five Gastrointestinal (GI) symptom clusters that depict reflux, abdominal pain, indigestion, diarrhea and constipation [25]. Among the GSRS, we used the digestion component portion, which consists of seven items, abdominal pains, heartburn, acid regurgitation, sucking sensations in the epigastrium, nausea and vomiting, borborygmus, abdominal distension and eructation.

### Statistical analysis

We selected gender, age, marital status, education level and lifestyle (tobacco use, alcohol use, physical activity and obesity) as covariates because we could obtain this information from our data based on previously published studies of the risk factors for sleep disturbances [26–29]. Baseline characteristics were summarized using descriptive statistics such as the proportion, mean and standard deviation. Continuous variables were categorized as appropriate categorical variables. The association between several factors and sleep disturbance was investigated using logistic regression after adjusting for confounding factors, then analyzed using two models. Model 1 was adjusted for demographic characteristics (gender, age, marital status, education level, tobacco use, alcohol use, physical activity and obesity) and total score of GSRS digestion. Model 2 was adjusted for demographic characteristics and seven items of GSRS digestion (abdominal pains, heartburn, acid regurgitation, sucking sensations in the epigastrium, nausea and vomiting, borborygmus, abdominal distension and eructation). A two-sided *p*-value < 0.05 was considered to indicate statistical significance. All data manipulations and statistical analyses were performed using Stata/MP version 15 (StataCorp LP, College Station, TX, USA).

## Results

### Demographic characteristics of participants

**Fig. 1:**
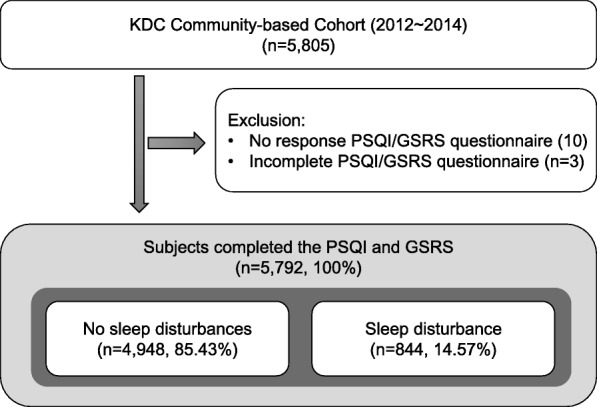
Selection of the cohort population. Among the participants, 5792 completed the Pittsburgh Sleep Quality Index (PSQI) and the Gastrointestinal Symptom Rating Scale (GSRS)

The total number of participants in the KDC community-based survey from 2012 to 2014 was 5805, all of whom signed an informed consent form. Among the participants, 5792 completed the Pittsburgh Sleep Quality Index (PSQI) and the Gastrointestinal Symptom Rating Scale (GSRS) (Fig. 1). The mean PSQI global score of the no sleep disturbances group (*n* = 4948) was 3.92 (SD = 2.14), while that of the sleep disturbance group (*n* = 844) was 11.18 (SD = 2.17). Demographic information describing the participants is presented in Table 1. Overall, 67.77 and 76.66% of the sleep disturbance group were female and married, respectively. (Table 1).

**Table 1:** Demographic characteristics of 5,792 subjects

	No sleep disturbances (*n* = 4,948)		Sleep disturbance (*n* = 844)		*p*
	*n*	%	*n*	%	
Gender					< 0.001
Male	2,418	48.87	272	32.23	
Female	2,530	51.13	572	67.77	
Age (year)					
Mean (SD)	61.99	9.64	64.06	10.15	< 0.001
< 65	3,180	64.27	459	54.38	< 0.001
65≤	1,768	35.73	385	45.62	
Marital status					< 0.001
Spouseless^a^	696	14.07	197	23.34	
Married	4,252	85.93	647	76.66	
Education level					< 0.001
Elementary school (≤6 years)	1,649	33.33	380	45.02	
Middle school (6-9 years)	990	20.01	178	21.09	
High school or above (≥9 years)	2,309	46.67	286	33.89	
Tobacco					< 0.001
No	4,274	86.38	767	90.88	
Yes	647	13.62	77	9.12	
Alcohol					0.001
No	2,842	57.44	537	63.6	
Yes	2,106	42.56	307	36.37	
Physical activity					0.005
No	1,749	35.10	341	40.40	
Yes	3,199	64.65	503	59.60	
Obesity^b^					0.468
No	2,883	58.27	503	59.60	
Yes	2,065	41.73	341	40.40	

^a^Managers and professionals include managers, professionals, technicians and associate professionals, clerical support workers, and service and sales workers

^b^Obesity indicates a BMI of 25 or greater

### Correlation between digestive symptoms and sleep disturbances

The sleep disturbances group reported significantly more intense and frequent symptoms than the no sleep disturbances group for all GSRS digestion items (Table 2). Moreover the overall magnitude of the difference between the digestive symptoms and the sleep disturbances was moderate except for the nausea and vomiting symptoms.

**Table 2:** Gastrointestinal Symptom Rating Scale (GSRS)-digestive items of 5,792 subjects

	No sleep disturbances (*n* = 4,948)		Sleep disturbance (*n* = 844)		*p*	Effect Size (Cohen’s d)		
	Mean	SD	Mean	SD		Estimate	95% CI	
GSRS-total digestion score	0.34	1.01	0.78	1.49	< 0.001	−0.40	−0.48	−0.33
Abdominal pains	0.02	0.17	0.08	0.30	< 0.001	−0.28	−0.35	−0.21
Heartburn	0.87	0.29	0.15	0.41	< 0.001	− 0.23	− 0.30	− 0.15
Acid regurgitation	0.06	0.26	0.13	0.38	< 0.001	−0.24	− 0.32	− 0.17
Sucking sensations in the epigastrium	0.02	0.17	0.06	0.30	< 0.001	−0.19	−0.26	− 0.11
Nausea and vomiting	0.02	0.14	0.03	0.20	0.0043	−0.11	−0.18	− 0.03
Borborygmus	0.03	0.19	0.07	0.20	< 0.001	−0.19	−0.27	− 0.12
Abdominal distension	0.06	0.25	0.14	0.39	< 0.001	−0.30	−0.38	− 0.23
Eructation	0.05	0.24	0.13	0.35	< 0.001	−0.28	−0.35	− 0.21

### Factors associated with sleep disturbances

The association between digestive symptoms and sleep disturbances was evaluated by logistic regression after adjusting for cofounding factors. In model 1, sleep disturbances were associated with being female (aOR = 1.66, 95% CI = 1.41–1.96), age (aOR = 1.02, 95% CI = 1.01–1.03), marital status (aOR = 0.72, 95% CI = 0.60–0.88), high school or above (aOR = 0.79, 95% CI = 0.66–0.94) and total score of GSRS digestion (aOR = 1.29, 95% CI = 1.22–1.36). In model 2, sleep disturbances were associated with being female (aOR = 1.66, 95% CI = 1.41–1.97), age (aOR = 1.02, 95% CI = 1.01–1.03), marital status (aOR = 0.72, 95% CI = 0.59–0.87), high school or above (aOR = 0.79, 95% CI = 0.66–0.94) and four digestive symptoms: abdominal pains (aOR = 1.63, 95% CI = 1.19–2.25), acid regurgitation (aOR = 1.48, 95% CI = 1.17–1.86), abdominal distension (aOR = 1.80, 95% CI = 1.42–2.28), eructation (aOR = 1.59, 95% CI = 1.24–2.03) (Table 3).

**Table 3:** Factors associated with sleep disturbance

	Model 1			Model 2		
	aOR	95% CI	*p*	aOR	95% CI	*p*
Female	1.66	1.41–1.96	< 0.001	1.66	1.41–1.97	< 0.001
Age	1.02	1.01–1.03	< 0.001	1.02	1.01–1.03	< 0.001
Marital status	0.72	0.60–0.88	0.001	0.72	0.59–0.87	0.001
High school or above (≥9 years)	0.79	0.66–0.94	0.007	0.79	0.66–0.94	0.008
Total score of GSRS digestion	1.29	1.22–1.36	< 0.001			
Abdominal pains	–			1.63	1.19–2.25	0.003
Acid regurgitation	–			1.48	1.17–1.86	0.001
Abdominal distension	–			1.80	1.42–2.28	< 0.001
Eructation	–			1.59	1.24–2.03	< 0.001
constant	0.06	0.03–0.11	< 0.001	0.05	0.03–0.10	< 0.001

Model 1 - adjusted for gender, age, marital status, education level, tobacco, alcohol, physical activity, obesity and total score of GSRS digestion

Model 2 - adjusted for gender, age, marital status, education level, tobacco, alcohol, physical activity, obesity, abdominal pains, heartburn, acid regurgitation, sucking sensations in the epigastrium, nausea and vomiting, borborygmus, abdominal distension and eructation

## Discussion

In this study, we demonstrated that sleep disturbances were associated with digestive symptoms (abdominal pains, acid regurgitation, abdominal distension and eructation), with some being closely associated.

According to many previous studies, sleep disturbances comprise an independent disease and a risk factor for many other diseases, including GI disorders such as IBS, gastroesophageal reflux disease, gastric ulcer, and duodenal ulcer [11–14, 30–34]. Moreover, a few studies have attempted to identify the cause of the association between sleep disturbances and sereval diseases. For example, Reddy attempted to identify differences in GI symptoms among poor sleepers based on variations in genes and found that the brain-derived neurotrophic factor genotype may be related to the etiology of poor sleep quality and chronic abdominal pain [35]. In addition, sleep disturbances were significant predictors of somatic complaints [36].

Similar to our findings, recent studies have commonly regarded female and age as risk factors related to sleep disturbances [26, 27]. For example, Dalmases reported that sleep health in a European population was associated with increasing age and being female [37]. Additionally, Choi reported that the incidence of GI diseases among sleep disturbance patients was much higher in females, as well as individuals of older age and with higher income in a multivariable analysis conducted using the National Health Insurance Service–National Sample Cohort [30]. In contrast to our findings, Mullane showed that obesity and smoking were risk factors for sleep disturbances [38], while Miller found interactive effects between insomnia and alcohol use among young adult veterans [39]. Accordingly, much more research is needed to investigate the relationship between demographic factors and sleep disturbances.

It should be noted that it was difficult to compare our findings with those of previous studies because we could not locate prior investigations that examined the relationship between specific digestive symptoms and sleep disturbance. However, the specific digestive symptoms presented in patients with sleep disturbance may be related to stomach disharmony, and these can be helpful to diagnosis of disease patterns in Traditional Chinese and Korean medicine.

Several limitations need to be considered while interpreting the results of this study. First, our findings were based on an association between digestive symptoms and sleep disturbance. To accurately evaluate the causal relationships, longitudinal data should be assessed. Second, there is the potential for self-reporting bias because we used perceived digestive symptoms tools and sleep quality tools; however, the GSRS and PSQI are good tools that have been shown to be reliable and valid in many studies. Third, we did not determine why only certain digestive symptoms among several digestive symptoms may be related to poor sleep quality. Accordingly, additional research is needed to investigate the genes, pain, and other mechanisms of digestive symptoms and sleep disturbances.

## Conclusions

Digestive symptoms and sleep disturbances seem to be associative. These results will help medical professionals to effectively diagnose and manage patients with sleep disturbance. Furthermore, future studies using comprehensive longitudinal data should be conducted to confirm the results of the present study.

## Abbreviations

FGID: Functional Gastrointestinal Disorders
GERS: Gastroesophageal Reflux Symptoms
GI: Gastrointestinal
GSRS: Gastrointestinal Symptom Rating Scale
KDC: Korean Medicine Data Center
KoGES: Korean Genome and Epidemiology Study
PSQI: Pittsburgh Sleep Quality Index
